# Dual effects of family ritual communication on Chinese adolescents’ well-being: supportive and constraining mechanisms

**DOI:** 10.3389/fpsyg.2025.1523315

**Published:** 2025-04-07

**Authors:** Ban Zhibin, Qiu Li, Cai Yulin, Wu Chunxiaoyu

**Affiliations:** ^1^School of Literature, Journalism and Communication, South-Central Minzu University, Wuhan, China; ^2^School of Information Management, Wuhan University, Wuhan, China; ^3^School of Art Design and Communication, Wuhan Huaxia Institute of Technology, Wuhan, China; ^4^Journalism and Information Communication School, Huazhong University of Science and Technology, Wuhan, China

**Keywords:** family ritual communication, adolescents, well-being, family modernization, support and constraint

## Abstract

**Background:**

Adolescent well-being has garnered significant attention from researchers both domestically and internationally. Much research focuses on the connection between adolescent well-being and family dynamics. As the main source of social support, the family significantly influences adolescent well-being. Western studies have identified family ritual communication as a crucial factor that can exert both positive and negative effects on adolescent well-being. However, most studies are contextualized within Western societies, and their findings may not be directly applicable to Chinese social settings.

**Methods:**

This research employed a questionnaire survey method to examine the impact of family ritual communication on the well-being of Chinese adolescents. A pilot survey was conducted to validate the questionnaire’s design before administering the final version. Initially, 150 questionnaires were distributed, yielding 144 valid responses. Following reliability and validity testing, a formal survey was conducted involving 848 adolescents aged 14 to 18. After eliminating responses with overly regular patterns, 755 valid questionnaires were retained.

**Results:**

The study elucidated the mechanisms through which family ritual communication affects the subjective well-being of Chinese adolescents, summarizing these effects as a form of psychological empowerment characterized by “support and constraint.” Family ritual communication serves as a supportive element, enhancing adolescent well-being through two mediators: family intimacy and resilience. Conversely, this form of psychological empowerment can also constrain adolescent well-being due to intergenerational power dynamics. Notably, the interdependent self-concept did not moderate the relationship between family ritual communication and the adolescents’ subjective well-being.

## 1 Introduction

In contemporary society, depression and anxiety among adolescents are pressing concerns, exacerbated by academic burdens and social pressures. These issues have increasingly drawn research attention. Studies on adolescent well-being have identified three critical factors: personal characteristics (such as personality, self-esteem, and self-efficacy), social networks (including friendships and campus life), and family environment (encompassing economic status and parenting styles). In post-industrial societies, adolescents often engage in structured activities like schoolwork and socializing, which are essential for success in a competitive labor market. However, this focus can diminish the time they spend with family (Offer, 2013). Given the limited time available for family interactions, it is crucial to foster positive family relationships. Family ritual communication, which holds significant symbolic value and is prevalent in all families, plays a crucial role in influencing adolescent well-being. The media frequently urges parents to engage in family rituals, citing their potential to enhance well-being. While Western research has explored the impacts of family ritual communication on adolescent well-being, showing both positive and negative effects, these findings are predominantly based on Western contexts and may not translate directly to Chinese society. Notably, family ritual communication in China retains elements of traditional Chinese family etiquette, a blend of ancient customs and modern life. Moreover, there are distinct cultural differences in “family” values between China and Western countries, with significant variations in family power dynamics across different cultural contexts (Tamm et al., 2017). Traditional Chinese Confucianism emphasizes parental authority, positing that adolescents should act in accordance with their parents’ expectations, whereas Western parents encourage independent decision-making among teenagers (Peng, 2023). In contemporary China, however, adolescents increasingly demonstrate respect for elders rather than strict obedience to them (Wang and Anderson, 2014). Therefore, it is essential to investigate the influence of Chinese family ritual communication on adolescent well-being within the modern Chinese context.

## 2 Literature review

The concept of family ritual is multifaceted, encompassing practices ranging from the younger generation’s kowtow to elders during the Spring Festival, to family birthday celebrations, to annual summer vacations. Since the 1950s, Western scholars have been examining the social phenomenon of family ritual. Wolin and Bennett (1984) characterized family ritual as a symbolic form of communication that provides satisfaction through repetitive activities.

Family rituals extend beyond mere behaviors; they are also communicative activities. Carey (1989) proposed the “Ritual View of Communication,” innovatively connecting communication with ritual, which significantly influenced the field. Rothenbuhler (1998) argued that “all rituals have communicative properties, and without a communicative effect, rituals are pointless.” This perspective is grounded in the idea that rituals consist of symbolic signs and embody a meaningful system. The most vital attribute of rituals is that the symbolic meanings embedded in the behaviors transcend the actions themselves. The purpose of family rituals is not for their direct function, but for the spiritual significance they aim at. For example, the main purpose of eating cake on a birthday is not nutritional but to convey blessings; similarly, allowing an elder to sit and eat first at a meal expresses respect and filial piety. General symbols establish connections between concrete objects and abstract concepts. In contrast, family rituals involve a secondary abstraction process that links two abstract concepts through homophonic words, mythological narratives, or cultural traditions. For example, in Northern China, eating dumplings during the Chinese New Year symbolizes “jiao zi” or the transition to a new year, representing a departure from the old and an embrace of the new.

While general symbols link concrete objects to abstract concepts, family rituals connect two abstract ideas, often bridged by homophonic words, mythological stories, or traditional customs. This research posits that family ritual communication, characterized by formal, repetitive, symbolic, and emotionally significant interactions among family members, lays the groundwork for strengthening familial bonds. The profound connection between adolescents and their families, as a familial factor, is also crucial to adolescents’ well-being. Empirical studies from the West indicate that family ritual communication can enhance feelings of belonging, cohesion, satisfaction, and well-being among family members, especially adolescents (Simões and Alberto, 2019; Kim and Jahng, 2019; Malaquias et al., 2015). However, some scholars contend that family rituals can also have adverse effects, such as creating additional burdens (Meske et al., 1994) and inciting conflicts (Leach and Braithwaite, 1996). Based on these considerations, the first hypothesis is proposed:

H1: The greater the intensity of family ritual communication, the stronger the subjective well-being of adolescents.

### 2.1 Family intimacy

Family intimacy refers to the emotional bond between family members and serves as a comprehensive indicator of closeness and a positive familial atmosphere. It represents the balance between autonomy and connection within the family. Numerous empirical studies have demonstrated that family ritual communication plays a crucial role in enhancing this intimacy. Ernest and Harvey (1948) posited that family rituals are among the eight critical factors influencing familial relationships, with richer rituals correlating with closer bonds. Segal (2004) highlighted that these rituals are symbolic activities that reinforce the identities and sense of belonging among family members. For example, communal meals are recognized not merely as food sharing occasions but as opportunities to strengthen familial bonds through communication. Such gatherings are vital rituals and primary social events that allow adolescents and their parents to converse, share emotions, and offer mutual support, thus reinforcing family unity (Páez et al., 2011). Spagnola and Fiese (2007) asserted that family rituals fortify attachments and connections among family members, establish strong emotional ties, create a family culture or identity, and foster positive emotions, which are particularly significant for family well-being. Marks et al. (2018) examined the influence of Sabbath rituals in Jewish families on their relational dynamics and found that these rituals unify family members and empower children. Conversely, the disruption of such rituals, such as families no longer dining together, can diminish the ceremonial significance of these gatherings and potentially weaken family cohesion. Therefore, the communication surrounding family rituals may affect adolescent well-being through the pathway of family intimacy. Accordingly, hypotheses 2–4 were proposed:

H2: The higher the intensity of family ritual communication, the greater the family intimacy among adolescents.

H3: The greater the family intimacy, the stronger the subjective well-being of adolescents.

H4: The intensity of family ritual communication positively influences the subjective well-being of adolescents through family intimacy.

### 2.2 Mediating effect of resilience

Resilience is an ability to withstand challenges such as substance abuse, mental health issues, or criminal behaviors, particularly under significant stress and difficulty (Linquanti, 1992). Individuals respond differently under pressure; some may withdraw, while others confront challenges directly. These varying coping strategies significantly affect their well-being.

Resilience is crucial not only in adverse situations like serious illnesses, bereavement, or divorce but also in everyday family life. It plays a beneficial role even without trauma or severe stress, promoting the well-being of family members, particularly adolescents navigating their formative years. Adolescents face substantial academic pressures and are beginning to navigate personal endeavors in love and career independently, often encountering setbacks that can heighten negative emotions and diminish their subjective well-being.

Resilience serves as a vital protective factor that enhances adolescent well-being. Studies, including those by Mahdian and Ghaffari (2016) and Yoon et al. (2015), have demonstrated the positive effects of resilience, finding that social support and family rituals, like shared dinners, provide stability and identity, thereby fostering resilience.

Family ritual communication can mitigate the negative effects of parental stress on children’s psychological health. Rahal et al. (2024) noted that frequent family gatherings could reduce drug abuse among young women. Curtiss (2018) observed that family rituals could ameliorate conditions in children with autism. Carla Crespo et al. (2013) investigated the role of family rituals in chronic disease management, revealing that such rituals offer emotional support and improve patient conditions. Conversely, disruptions in family rituals due to external factors, such as parental alcohol abuse, can adversely affect adolescent development. Hawkins (1997) found that interruptions in family rituals, like missing family dinners due to parental alcohol abuse, are associated with increased likelihood of drug abuse, antisocial behavior, and psychological issues like depression and anxiety in children. Consequently, hypotheses 5–7 were proposed:

H5: The greater the intensity of family ritual communication, the higher the resilience of adolescents.

H6: The stronger the resilience of adolescents, the stronger their subjective well-being.

H7: The intensity of family ritual communication positively impacts the subjective well-being of adolescents through resilience.

### 2.3 The mediating effect of cultural identity

Berry (2005) posited that cultural identity is the cognitive and emotional attachment individuals in different cultural communities have to their own and other cultures. The adoption of shared cultural symbols, including language and ritual symbols, is a crucial foundation for cultural identity. In pre-modern societies, cultural identity was less of a concern due to low population mobility and a closed social structure. However, as modernization has intensified, the blending and clashing of Eastern and Western cultures have made cultural identity a topic of high interest, closely linked to family ritual communication.

Family ritual communication enhances cultural identity, as rituals often embody cultural traditions. Historically, rituals have been acknowledged as vital for the cohesion of human groups; Durkheim considered them a central element of his theory of mechanical solidarity. In both modern and traditional settings, rituals form a critical component. Social rituals are symbolic behaviors developed within a group and are repeated for their inherent meaning and the satisfaction derived by participants, even though these participants might not always articulate the benefits or functions explicitly, as these functions are not purely instrumental (Klapp, 1959). Moreover, an individual’s identification with their group can influence their well-being. TIdentity theory suggests that group identity is a significant source of self-esteem and fulfills psychological needs such as belonging, both of which are vital for well-being. Cultural identity, as a key aspect of social identity, also affects an individual’s psychological perceptions. Individuals recognize their affiliation with certain groups, and this identification imbues them with specific emotions and values, deriving not only from familial ties but also from the broader cultural identity of the group. Usborne and Taylor (2010) found a positive correlation between the clarity of cultural identity and personal identity, self-esteem, and psychological well-being. While Chinese civilization emphasizes a collectivist culture, individualistic culture dominates in Western societies. Diener et al. (1995) observed that in individualistic countries, overall well-being—especially marital satisfaction—is high, though these countries also exhibit elevated divorce and suicide rates. Additionally, recent research by Zhou et al. (2023) identified a positive correlation between red cultural identity and subjective well-being within the Chinese cultural context. These findings illustrate that the cultural identity of one’s ethnic group significantly influences their subjective well-being. Accordingly, hypotheses 8–10 were proposed:

H8: The greater the intensity of family ritual communication, the higher the adolescents' recognition of traditional culture.

H9: The stronger the traditional cultural identity of adolescents, the stronger their subjective well-being.

H10: The intensity of family ritual communication positively impacts the subjective well-being of adolescents through cultural identity.

### 2.4 The regulatory effect of interdependent self-concept

In cross-cultural research, the concepts of collectivism and individualism are commonly used to distinguish cultural differences between the East and the West. Markus and Kitayama (1991) identified two types of self-concept at the personal level: interdependent and independent. Individuals with a strong independent self-concept tend to emphasize their distinctiveness and unique characteristics when describing “who I am.” Conversely, those with a strong interdependent self-concept often describe their identity in terms of their relationships, such as being someone’s daughter or a member of a specific group. In Western cultures, the independent self-concept prevails, influencing behavior based on personal thoughts and feelings rather than those of others. In Eastern cultures, interdependence dominates, and there is a greater reliance on communal relationships. Scholars, including Lu et al. (2001), have noted significant differences in well-being between Chinese and Western populations, attributing these differences to interdependent self-construal. Typically, in individualistic cultures, the focus is on personal pleasure, whereas in collectivist cultures, maintaining harmonious interpersonal relationships is deemed crucial. This finding is supported by extensive empirical research; for example, McMahon (2004) confirmed that harmonious relationships significantly influence well-being in collectivist settings. In individualistic societies, well-being is often associated with pleasure, vitality, and self-actualization. Cross-cultural studies, such as those by Kwan et al. (1997) and his team, have shown that harmonious interpersonal relationships more profoundly affect well-being in collectivist cultures, such as in Hong Kong, compared to individualistic ones, like the U.S. Lam et al. (2012) explored perceptions of harmony, well-being, and health among Hong Kong families, identifying harmony as a central element of family functioning. However, individualism and collectivism are not mutually exclusive. Particularly in today’s globalized world, it is oversimplified to assume that all Westerners subscribe to individualism, just as not all East Asians, influenced by Confucian culture, adhere strictly to collectivism. Steele and Lynch (2013) proposed that with China’s social transformation and economic development, individualistic elements are increasingly influencing subjective well-being. Based on these observations, hypothesis 11 was proposed:

H11: Self-concept modulates the impact of family ritual communication on adolescents' well-being; specifically, the stronger the self-concept, the greater the impact of family ritual communication on adolescents’ well-being.

**Figure 1:**
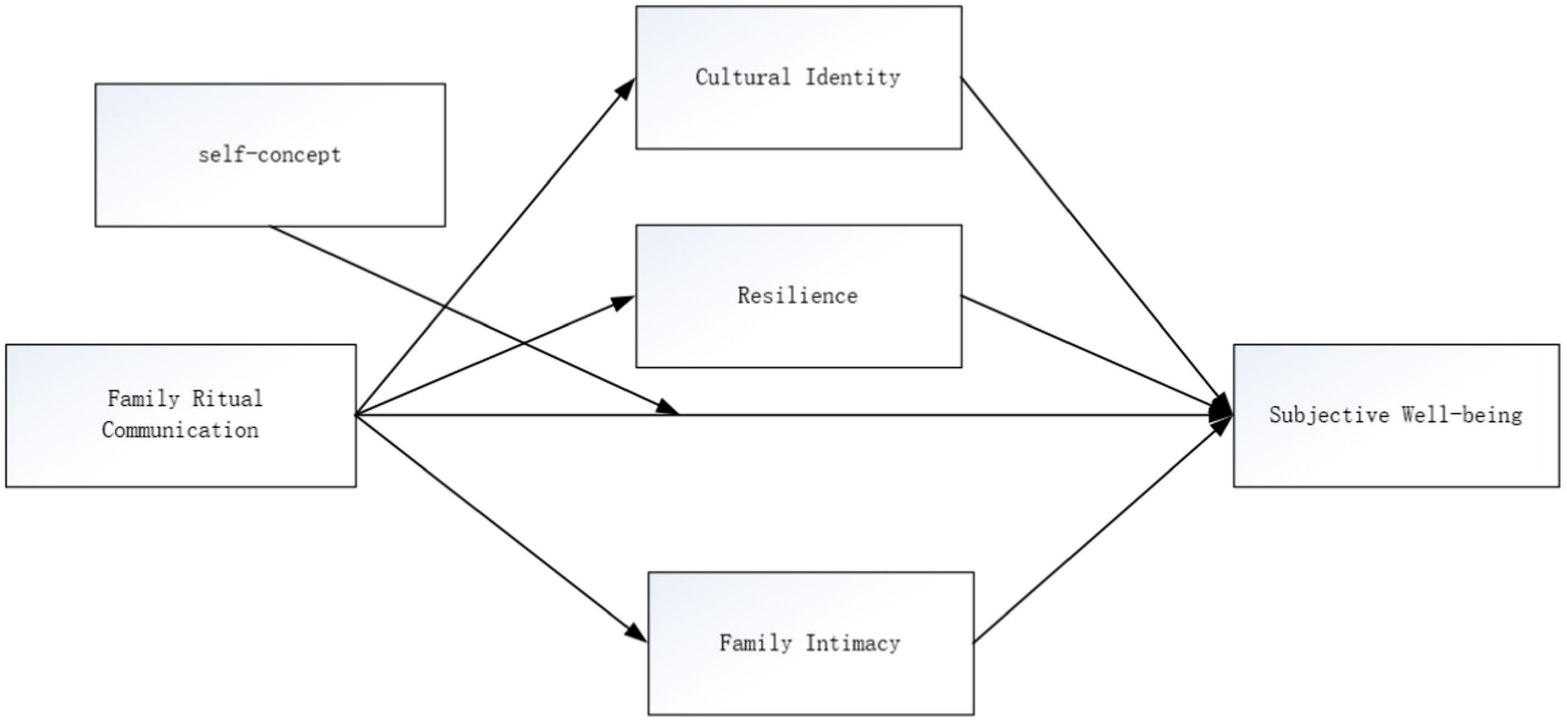
Theoretical model of the impact of Chinese family ritual communication on adolescents’ well-being.

In summary, the conceptual model constructed in this research is illustrated in Figure 1.

## 3 Materials and methods

### 3.1 Questionnaire design

The variable of family ritual communication is assessed using the Family Ritual Questionnaire, developed by Fiese and Kline (1993), which examines the intensity of family ritual communication. This study focuses on daily interactive rituals, festival celebrations, significant events, and family etiquette. Each category is evaluated across four dimensions: occurrence, symbolic meaning, emotion, and pattern, comprising a total of 16 items rated on a 5-point Likert scale (ranging from 1 = strongly disagree to 5 = strongly agree). The Chinese version of the Family Environment Scale (FES-CV), adapted by Fei Lipeng, measures family intimacy. Originally developed by Moss, this scale was revised by Wang X., et al. to suit Chinese contexts; however, only items pertinent to family intimacy were selected for this study. Subjective well-being is measured according to the framework proposed by psychology professor Diener (1984) at the University of Illinois, encompassing three dimensions: positive emotions, negative emotions, and life satisfaction. The Bradburn Emotion Scale, used to assess emotional states, includes five statements each on positive (e.g., “feeling comfortable and happy in current life”) and negative emotions (e.g., “often feeling sad and depressed”). Life satisfaction is gauged using Diener’s Life Satisfaction Scale, which includes items such as “My life is close to the ideal state” (Diener et al., 1985). Resilience is measured using the scale developed by Connor and Davidson (2003), featuring 10 statements such as “I can adapt when things change.” Cultural identity is assessed using the Ethnic Identity Measurement Instrument (MEIM) developed by Phinney (1992), and the improved Cultural Identity Scale by Ran Hua, which includes 12 statements like “I am proud to be a member of the Chinese nation.” Self-concept is measured using the English Self-Concept Scale by Singelis (1994), which assesses independent and interdependent self-concepts, including 12 questions on interdependent self-construction. The Chinese version, translated and adapted by Hou Yu in her doctoral thesis (Hou, 2020), includes five statements such as “For me, maintaining a harmonious relationship with others is very important” and was validated for its reliability.

### 3.2 Data collection

A pilot survey was conducted to verify the questionnaire’s rationality before distributing the final version. Out of 150 distributed questionnaires, 144 were returned, resulting in an effective response rate of 96%. The main survey targeted the impact of family ritual communication on the well-being of Chinese adolescents. It involved 848 adolescents aged 14 to 18, excluding responses with overly regular patterns. A total of 755 valid questionnaires were collected, yielding an effective response rate of 89.03%. The gender distribution among participants was nearly balanced, with 403 females (53.4%) and 352 males (46.6%).

## 4 Results

### 4.1 Test of reliability and validity

Testing the reliability and validity of the scale is essential to ensure its stability and accuracy. Reliability tests employ Cronbach’s ɑ coefficients of the overall scale and each variable, assessing the correlation between each item and the total score. According to Wu Minglong, a Cronbach’s ɑ coefficient above 0.8 indicates high reliability and internal consistency. The overall Cronbach’s α for the questionnaire was 0.968, with each of the eight variables including family ritual communication and resilience exceeding 0.85. Additionally, composite reliability (CR) values were above 0.7, confirming the questionnaire’s reliability. The Kaiser-Meyer-Olkin (KMO) values for these variables ranged from 0.843 to 0.961, and the Bartlett’s test of sphericity returned a *p*-value of <0.001, validating the data’s suitability for factor analysis. Principal component analysis extracted common factors with eigenvalues over 1, resulting in a cumulative variance contribution rate of 80.318%. Maximum variance rotation confirmed that each item had a loading greater than 0.40 on the common factors, demonstrating good construct validity.

### 4.2 Overall fitting degree of structural equation model

This research used Amos23.0 to analyze the fitting of the structural equation model. Key indicators included a chi-square to degrees of freedom ratio of 4.171, which is less than 5.0; GFI of 0.867, CFI of 0.942, TLI of 0.932—all exceeding 0.8; and RMSEA of 0.073, which is less than 0.08. These results indicate a satisfactory fit of the model to the data, confirming the acceptability of this theoretical model.

### 4.3 Test of mediating and moderating effects

A mediation effect model was developed with well-being as the dependent variable, family ritual communication as the independent variable, and family intimacy, resilience, and cultural identity as mediating variables. Interdependent self-concept was analyzed as a moderating variable using PROCESS Model 5, Bootstrap with a 95% confidence interval (CI). Validation was performed using the percentile method, and results are displayed in Tables 1 and 2.

**Table 1:** Results of mediating effect test.

Index	Cultural identity	Resilience	Family intimacy	Family well-being	Family well-being
Family ceremony communication	0.445 (<0.001)	0.517 (<0.001)	0.695 (<0.001)	0.365 (<0.001)	0.087 (0.020)
Cultural identity					−0.131 (<0.001)
Resilience					0.353 (<0.001)
Family intimacy					0.350 (<0.001)
Interdependent self-concept					0.148 (<0.001)
Family ritual communication * interdependent self-concept				0.355 (<0.001)	0.019(0.409)
Adjusted R formula	0.198	0.267	0.483	0.369	0.514
*F* (*p*-value)	185.976 (<0.001)	273.952 (<.001)	703.234 (<0.001)	219.641 (<0.001)	131.966 (<0.001)

The data had been standardized; The *p*-value was in parentheses. The symbol (*) represents the product of the independent variable and the moderator variable.

**Table 2:** Decomposition of mediating effects of family ritual communication on well-being.

Hypothesis	Route	Bootstrap 95% confidence interval	The value of indirect effects	The proportion of indirect effects to direct effects
H10	① Family ritual communication → cultural identity → sense of well-being	[−0.069, −0.015]	−0.040	62.25%
H7	② Family ritual communication → resilience → well-being	[0.121, 0.194]	0.155	238.52%
H4	③ Family ritual communication → Family intimacy → well-being	[0.154, 0.248]	0.203	312.17%
	Sum of intermediary effects	② + ③	0.357	550.08%
	Masking effect	①	−0.040	62.25%

According to Table 1, the direct effect of family ritual communication on adolescents’ subjective well-being was statistically significant (*p* < 0.05) and positive. However, the interaction term coefficient for interdependent self-concept was 0.019, with a *p*-value of 0.409, indicating no significant moderating effect.

As shown in Table 2, the mediating effect of cultural identity between family ritual communication and well-being was −0.040, with a 95% confidence interval of [−0.069, −0.015], excluding 0. This indicates a significant negative mediating effect, contrary to the direct effect. The resilience mediating effect was 0.155, with a confidence interval of [0.121, 0.194], also excluding 0, signifying a substantial mediating effect. The mediating effect of family intimacy was even stronger at 0.203, with a confidence interval of [0.154, 0.248], further excluding zero, indicating a significant positive mediating influence. These results support Hypothesis H4, suggesting family ritual communication positively impacts well-being through family intimacy and resilience, but negatively through cultural identity. The order of mediation effect magnitude was: family ritual communication → family intimacy → well-being>family ritual communication → resilience → well-being>family ritual communication → cultural identity → well-being.

### 4.4 Summary of hypothesis test results

**Figure 2:**
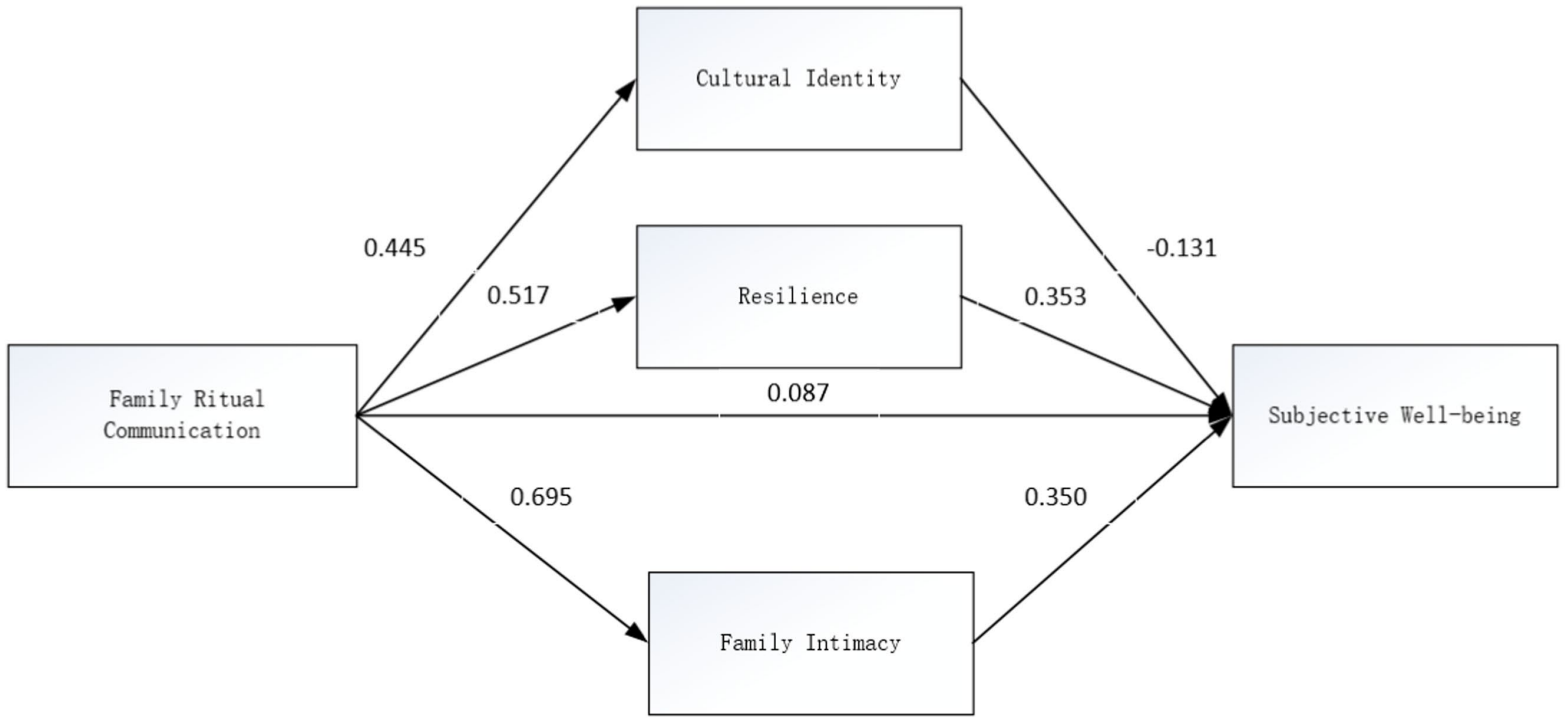
Parallel mediation model of family intimacy, resilience, and cultural identity.

The mechanisms underlying the impact of family ritual communication on adolescents’ subjective well-being were elucidated through mediating and moderating effects analysis. The study confirmed that family ritual communication positively influences adolescents’ well-being, mediated by family intimacy and resilience, which both positively affect well-being, whereas cultural identity exerts a negative effect. Additionally, no moderating effect of interdependent self-concept was observed. These findings, including three mediating pathways—resilience, family intimacy, and cultural identity—highlight that family intimacy has the most substantial mediating effect at 0.203, followed by resilience at 0.155. The negative mediating effect of cultural identity was −0.040. Results are detailed in Table 3, and a structural equation model was constructed for path analysis, presented in Figure 2.

**Table 3:** Comparison of Hypothesis Test Results.

Hypothesis number	Brief content of Hypothesis	Results
H1	Family ritual communication → well-being	Supporting
H2	Family ritual communication → Family intimacy	Supporting
H3	Family intimacy → well-being	Supporting
H4	Family ritual → Family intimacy → well-being	Supporting
H5	family ritual communication → resilience	Supporting
H6	Resilience → well-being	Supporting
H7	Family ritual communication → resilience → well-being	Supporting
H8	family ritual communication → cultural identity	Constrained
H9	Cultural identity → well-being	Constrained
H10	Family ritual communication → cultural identity → well-being	Supported
H11	Family ritual communication* interdependent self-concept → sense of well-being	Non-supported

The symbol (*) represents the product of the independent variable and the moderator variable.

## 5 Research conclusion and discussion

### 5.1 Conclusion

#### 5.1.1 Mediating effect of family intimacy

Family intimacy is a crucial component of the adolescent growth environment, enhancing their positive emotions. The analysis revealed that family ritual communication, which fosters emotional bonds between adolescents and their family members, positively impacts adolescent well-being through family intimacy. Simões and Alberto (2019) asserted that the primary function of family ritual communication is to forge connections, initially with family members and subsequently with ancestral values, culture, traditions, and religion. It links nuclear families to broader familial contexts, fostering intimacy and unity, which are essential for constructing and transmitting family identity. Li (2018) observed that economic necessities often require family members to live apart, presenting significant challenges to maintaining family intimacy. Family ritual communication compensates for this separation by helping to readjust family roles, sustain collective consciousness, facilitate family reunions, transmit family values, and ultimately enhance family identity recognition and intimacy. This study supports these findings, demonstrating that family rituals provide a vital platform for interaction, especially when busy schedules and limited time reduce opportunities for family gatherings. Additionally, family ritual communication strengthens bonds between relatives, as family gatherings during events like the Chinese Spring Festival consolidate these relationships. Despite hectic work schedules, individuals prioritize returning home for the reunion dinner and visiting relatives, which not only maintains familial bonds but also enhances the festive atmosphere and interaction among family members.

#### 5.1.2 Mediating effect of resilience

Susana Santos posited that family ritual communication plays a critical role in alleviating the emotional distress caused by family stresses and changes. It serves several functions: firstly, it offers a respite from distress, as activities like regular family travels provide relaxation. Secondly, it fosters a sense of security, emotionally shielding family members. Thirdly, it instills hope, encompassing positive future expectations. Lastly, it helps individuals focus on the essence of life rather than on familial hardships, such as illnesses. Therefore, family ritual communication aids families in navigating challenges (Santos et al., 2018). For adolescents, the symbolic aspects of family rituals can lessen their distress during difficult times. For example, funeral ceremonies can mitigate the grief associated with the loss of a loved one. Additionally, family ritual communication can inspire positive self-affirmations linked to simple folk beliefs. In traditional Chinese culture, these beliefs—considered a form of universal religion—include faith in celestial and ancestral deities. During stressful periods, these spiritual beliefs can significantly reduce perceived stress and enhance well-being.

#### 5.1.3 The masking effect of cultural identity

Data analysis revealed that cultural identity exerts a masking effect on the impact of family ritual communication on adolescents’ well-being. The term ‘masking effect’ originally described how external interferences weaken sensory perceptions. In academic contexts, it refers to the diminution of the primary effect between two variables by a “masking variable.” This study found that cultural identity moderates the impact of family ritual communication on adolescents’ well-being. While family ritual communication positively affects cultural identity, this identity, in turn, can diminish the positive influence of family rituals on adolescent well-being. On one hand, family rituals bolster adolescents’ cultural identity; on the other, compelling adolescents to embrace the symbolic meanings of these rituals can adversely affect their sense of well-being. Coffelt (2018) found that although family rituals enhance cohesion, economic constraints or family disputes might prevent young members from relating to the rituals’ conveyed meanings (Fiese, 1992), leading them to disengage and label these rituals as empty. This disconnection is also prevalent in Chinese families, where traditional values often prioritize family interests over individual desires. Despite the erosion of this traditional value system, parental authority persists. Adolescents, often influenced by new concepts through work, study, and social media, are compelled to participate in traditional family rituals with which they may not identify. This enforced participation can evoke negative emotions, making adolescents feel alienated and disconnected from their family (Malaquias et al., 2015).

#### 5.1.4 Limitations of the research

This research explores the mechanisms through which family ritual communication impacts adolescents’ well-being, identifying three mediating pathways: family intimacy, resilience, and cultural identity. However, there remains significant room for improvement. Future studies should investigate additional intermediary pathways with further literature support and data verification. A more comprehensive understanding of the interaction processes between parents and adolescents during family ritual communication is needed, along with data collection from parents in addition to adolescents, to deepen future research.

### 5.2 Discussion

Adolescence is a period marked by heightened risk for depression and anxiety, attributable to both external and internal factors. Externally, adolescents face high expectations and intense competition in preparation for their future careers. Internally, significant physical and psychological changes occur during this period, prompting studies on how to enhance their subjective well-being at home and foster positive interpersonal relationships. Previous research has examined factors affecting adolescent family well-being, including parental marital quality, family economic status, family structure, and parenting style. While certain aspects of family ritual communication, such as beliefs in filial piety, are recognized to positively correlate with adolescents’ family well-being, the impact of family rituals has not been extensively explored through the lens of ritual communication. This research incorporated family ritual communication as a factor influencing adolescent well-being and found it supportive, positively impacting well-being through two mediating pathways: family intimacy and resilience. Family intimacy is enhanced by shared activities such as nightly dinners, regular travel, and celebrating birthdays, which contribute to beautiful memories and closer family bonds. Resilience is bolstered as adolescents gain a sense of security from family rituals, equipping them to face future challenges with strength.

However, the study also identified that within the context of traditional family authority, family ritual communication can negatively affect adolescent well-being. Empirical findings suggest that cultural identity acts as a masking variable, diminishing the positive effects of family ritual communication on well-being. Due to generational differences, the forms of family ritual communication favored by older generations may not resonate with adolescents. In contexts driven by Chinese filial piety culture, adolescents are expected to participate and heed their elders in family rituals, even if they do not align with the rituals’ symbolic meanings, which can adversely affect their well-being.

This research found that family ritual communication can enhance the well-being of adolescents and provide psychological empowerment. However, this empowerment often occurs within the framework of intergenerational power dynamics, leading to a dual role of support and constraint in the psychological empowerment mechanism. This model breaks through traditional unidimensional explanatory frameworks, marking the first integration of facilitative factors and inhibitory factors into a unified analytical system. The “Supportive and Constraining Dual-Effects Model” reveals the dialectical unity of family ritual communication through two opposing mechanisms. On one hand, family ritual transmission constructs well-being via the accumulation of emotional energy and reproduction of symbolic meanings. On the other hand, conflicts between traditional cultural norms and modernity simultaneously suppress well-being. By mapping these complex mediating pathways, the Dual-Path Model provides a novel framework for understanding Chinese adolescents’ well-being, while contributing an Eastern perspective to global family communication research.

## Data Availability

The original contributions presented in the study are included in the article/supplementary material, further inquiries can be directed to the corresponding author.
